# Metabolic Impact of Light Phase-Restricted Fructose Consumption Is Linked to Changes in Hypothalamic AMPK Phosphorylation and Melatonin Production in Rats

**DOI:** 10.3390/nu9040332

**Published:** 2017-03-27

**Authors:** Juliana de Almeida Faria, Thiago Matos F. de Araújo, Daniela S. Razolli, Letícia Martins Ignácio-Souza, Dailson Nogueira Souza, Silvana Bordin, Gabriel Forato Anhê

**Affiliations:** 1 Department of Pharmacology, Faculty of Medical Sciences, State University of Campinas, #105 Alexander Fleming St., Campinas SP 13092-140, Brazil; ju.almeidafaria@gmail.com (J.d.A.F.); dailson.ns@gmail.com (D.N.S.); 2 Laboratory of Cell Signaling, Faculty of Medical Sciences, State University of Campinas, Carl von Linnaeus St., Campinas SP 13083-864, Brazil; thiagomatosaraujo@gmail.com (T.M.F.d.A.); danirazolli@yahoo.com.br (D.S.R.); 3 Faculty of Applied Sciences, State University of Campinas, #1300 Pedro Zaccaria St., Limeira SP 13484-350, Brazil; leticia.isouza@gmail.com; 4 Department of Physiology and Biophysics, Institute of Biomedical Sciences, University of Sao Paulo, Sao Paulo SP 05508-900, Brazil; silvana.bordin@gmail.com

**Keywords:** fructose, out-of-phase feeding, AMPK, corticosterone, melatonin

## Abstract

Recent studies show that the metabolic effects of fructose may vary depending on the phase of its consumption along with the light/dark cycle. Here, we investigated the metabolic outcomes of fructose consumption by rats during either the light (LPF) or the dark (DPF) phases of the light/dark cycle. This experimental approach was combined with other interventions, including restriction of chow availability to the dark phase, melatonin administration or intracerebroventricular inhibition of adenosine monophosphate-activated protein kinase (AMPK) with Compound C. LPF, but not DPF rats, exhibited increased hypothalamic AMPK phosphorylation, glucose intolerance, reduced urinary 6-sulfatoxymelatonin (6-S-Mel) (a metabolite of melatonin) and increased corticosterone levels. LPF, but not DPF rats, also exhibited increased chow ingestion during the light phase. The mentioned changes were blunted by Compound C. LPF rats subjected to dark phase-restricted feeding still exhibited increased hypothalamic AMPK phosphorylation but failed to develop the endocrine and metabolic changes. Moreover, melatonin administration to LPF rats reduced corticosterone and prevented glucose intolerance. Altogether, the present data suggests that consumption of fructose during the light phase results in out-of-phase feeding due to increased hypothalamic AMPK phosphorylation. This shift in spontaneous chow ingestion is responsible for the reduction of 6-S-Mel and glucose intolerance.

## 1. Introduction

Impaired glucose tolerance (defined for humans as 2 h values in the oral glucose tolerance test ranging between 140 mg/dL and 199 mg/dL) [1] and insulin resistance (resistance to insulin-stimulated glucose uptake) [2] can be induced in rodents and humans by excessive fructose consumption. For instance, Sprague–Dawley rats fed a fructose-enriched diet develop impaired glucose tolerance and whole body insulin resistance [3]. In mice, fructose-enriched diets were found to cause impaired glucose tolerance with concomitant hepatic triglyceride accumulation and insulin resistance [4]. Interventional experiments with humans have also demonstrated that overweight subjects display impaired glucose tolerance after a 10-week interval of consumption of fructose sweetened beverages [5]. Among the myriad of endocrine changes that putatively underlie these metabolic effects, the consumption of a fructose-enriched diet was shown to reduce nocturnal melatonin production in rats [6].

The relationship between melatonin and the control of energy metabolism has been supported by several studies using distinct experimental approaches. Surgical ablation of the pineal gland was reported to result in impaired glucose tolerance and insulin resistance with increased nocturnal levels of glycemia and gluconeogenesis [7,8,9]. Accordingly, the aging-related reduction of melatonin levels was shown to mediate the enhanced adiposity in middle-aged rats [10]. In turn, exogenous melatonin administration is able to improve the metabolic control in rodents rendered glucose intolerant either by high-fat diets or fructose administration [11,12,13].

Recent studies have revealed that metabolic outcomes caused by fructose and high-fat diet intake is influenced by their out-of-phase consumption. Mice allowed to consume a high-fat diet exclusively during the dark-phase fail to develop abrupt body weight gain and impaired glucose tolerance relative to those subjected to ad libitum or light phase-restricted consumption [14,15]. In addition, fructose consumption by mice exclusively during the light phase, but not during the dark phase, resulted in increased body weight, adiposity and insulin levels [16]. However, the precise mechanism by which fructose induces these changes are not known. We have previously demonstrated that short-term fructose injections in the central nervous system during the light phase leads to an increase in the endogenous glucose production (EGP) by hypothalamic AMP-activated protein kinase (AMPK) activation [17]. It was also shown that hypothalamic AMPK activation by fructose is also important to acutely stimulate food intake [18].

Depending on its intensity and frequency, out-of-phase food intake by humans can be classified as the night eating syndrome (NES) [19]. The prevalence of NES is relatively low in the general population but ranges between 8.9% and 27% in obese subgroups [20]. Cohort studies have shown that NES positively correlates with the diagnosis of metabolic syndrome, increased triglycerides and waist circumference [21]. Among several adaptations, the circadian endocrine profile of NES patients is characterized by reduced melatonin levels during the night and increased morning cortisol concentrations [22].

Given the mentioned observations, the present study was conducted to investigate whether the metabolic impact resulting from fructose consumption during different phases of the light/dark cycle is dependent on changes in hypothalamic AMPK activation and in the circadian pattern of food intake in rats. We also collected results suggesting that disruption of melatonin production is a key event in the mechanism linking the light phase-restricted fructose consumption and its metabolic outcomes.

## 2. Materials and Methods

### 2.1. Animals and Treatments

The experimental procedures were approved by the State University of Campinas Committee for Ethics in Animal Experimentation (protocol No. 3506-1) and were conducted in accordance with the guidelines of the Brazilian College for Animal Experimentation.

Three-week-old male Sprague–Dawley rats were obtained from the Animal Breeding Center at the University of Campinas (CEMIB, Campinas, Sao Paulo, Brazil) and were housed at 22 ± 2 °C under a 12:12 h light:dark cycle (lights on at 7:00 a.m.) with free access to food and water for 5 weeks. At 8 weeks of age, rats were assigned to the experimental groups for an additional 8 weeks of treatment with fructose and/or melatonin. Measurements of chow consumption and body mass were made twice and once a week during the period of treatment, respectively. When specified in the results section, one of the three experimental strategies was combined with the fructose treatment: (i) chow availability was restricted to the dark phase (Chow-R rats) during the 8 weeks of treatment; (ii) compound C was administered during the last week of treatment through a cannula placed in the lateral ventricle or (iii) melatonin dissolved in the drinking water was administrated during the dark phase during the 8 weeks of treatment.

Fructose was dissolved in regular water to produce a 70% stock solution (*w*/*v*). The fructose stock solution was further diluted to 10% (*w*/*v*) with regular water immediately before treatment. Fructose was made available exclusively during the light or the dark phases (LPF and DPF rats, respectively). Bottles containing just water were offered to the rats only when fructose was absent.

Melatonin (Cat. A9525; Sigma-Aldrich, St. Louis, MO, USA) was initially diluted in 100% ethanol to generate a 100 mg/mL stock solution that was kept protected from the light in −20 °C for no longer than 7 days. The melatonin stock solution was further diluted (1:50) with distilled water every 3 days of treatment to generate a 2 mg/mL solution. Variable volumes of this solution were added to the drinking water bottle to yield a 0.5 mg/kg ingestion of melatonin. Bottles with melatonin were placed on the cages 30 min before “lights off” and removed 30 min after “lights on”. The individual calculations of the required volumes of melatonin solution were based on the body weight (weekly assessed) and nocturnal water intake (daily assessed) and were adjusted daily.

### 2.2. Surgical Procedure and Intracerebroventricular Treatment

Six weeks after the beginning of treatment using fructose solution, rats were anesthetized with diazepam and ketamine (2 and 50 mg/kg, respectively) and placed in a stereotaxic apparatus to insert a stainless-steel cannula into the lateral ventricle. Stereotaxic coordinates were 0.8 mm (anteroposterior), 1.5 mm (lateral), and 4.0 mm (depth) [23]. The localization of the cannula was tested by evaluating the dipsogenic response to an intracerebroventricular (icv) angiotensin II injection (5 ng/μL saline; Sigma, St. Louis, MO, USA) 1 week after the surgical procedure (7th week of fructose treatment). Only animals that presented a positive response in these tests were used for further experimentation.

The pharmacological inhibitor of AMPK, Compound C (Cat. 171260; EMD4 Biosciences, Gibbstown, NJ, USA) was diluted in 5% dimethylsulfoxide (DMSO) to a final concentration of 200 mM and was injected daily through the cannula for five days during the last week of treatment with fructose. Injections (2 μL) were performed 1 h after lights on Zeitgeber Time 1 (ZT 1) and equal volumes of vehicle were injected in the controls. Experiments with these rats were carried out two hours before lights off (ZT 10) on the day of the last icv injection. Fasting prior to the analyses and sample collection started immediately after the last injection (between ZT 1 and ZT 2).

### 2.3. Intraperitoneal Pyruvate Tolerance Test (pTT)

Rats were fasted for 10 h, and a sodium pyruvate solution (250 mg/mL) was injected intraperitoneal (i.p.) at a dosage of 2 g/kg. Pyruvate injections were made two hours before lights off. Glucose concentration was determined in blood extracted from the tail before (0 min) and 15, 30, 60, 90, and 120 min after pyruvate injection. The area under the curve (AUC) of glycemia vs. time was calculated using each individual baseline (basal glycemia) to estimate glucose clearance after pyruvate injection.

### 2.4. Intraperitoneal Glucose Tolerance Test (GTT)

Rats were fasted for 10 h prior to i.p. glucose injection (2 g/kg of a 25% solution of d-glucose) two hours before lights off. The blood samples were collected from the tail at 0, 10, 15, 30, 60 and 120 min to determine the blood glucose concentration. The area under the curve (AUC) of glycemia vs. time was calculated from each individual baseline (basal glycemia) to estimate glucose tolerance.

### 2.5. Intraperitoneal Insulin Tolerance Test (ITT)

Rats were fasted for 10 h prior to i.p. insulin injection (2 IU/kg) two hours before “lights off”. Blood glucose was measured before and 5, 10, 15, 20, 25 and 30 min after insulin injection to determine the sensitivity of insulin-responsive tissues. Blood glucose values were converted to a logarithmic scale, and the slope of the curve was calculated. This value multiplied by 100 was assumed to be the glucose decay constant (KITT).

### 2.6. Protein Extraction and Immunoblotting

Anesthetized rats were decapitated, and the hypothalamus was removed and processed for Western blotting as previously described [17]. The hypothalamic tissue removed for Western blot analysis contained approximately 4.0 mm^3^ and had the optic chiasm as the rostral limit (bregma −0.25 mm), the infundibular stem as the caudal limit (bregma −4.20 mm) and were 4.0 mm wide and 3.0 mm deep [24]. The extractions occurred two hours before lights off (ZT 10). The primary antibodies used were as follows: anti-pAMPK alpha (T172) from Cell Signaling Technology (Cat. 2531S, Danvers, MA, USA) and anti-GAPDH from Cell Signaling (Cat. 2118S, Danvers, MA, USA). Secondary antibodies conjugated with horseradish peroxidase (Bio-Rad Laboratories, Hercules, CA, USA) were used, followed by chemiluminescent detection of the bands on X-ray-sensitive films. Optical densitometry was performed using the Scion Image analysis software (version, Scion Corp., Frederick, MD, USA).

### 2.7. Immunofluorescent Staining

Initial perfusion of the anesthetized rats with saline was followed by perfusion with 4% paraformaldehyde. After perfusion, the animals were decapitated and they had the encephalon excised. Each fixed encephalon was cut into a fragment limited by the bregma −0.25 mm (rostral) and bregma −4.20 mm (caudal). The sections (5.0 micrometer thick) used for staining were between bregma −2.50 and −2.80. The atrium of the central, third and lateral ventricles as well as the hippocampus served as indicators for the sections within this antero-posterior interval [24]. Sections were incubated with primary antibody against pAMPK (Thr 172) (Cat. 2535, Cell Signaling Technology, Danvers, MA, USA) overnight at −4 °C and with secondary antibody conjugated to Alexafluor 546 for 2 h (Cat. A10040, Thermo Fisher Scientific, Waltham, MA, USA). The 4′,6-diamidino-2-phenylindole (DAPI) stain (Cat. H-1200, Vector Laboratories, Burlingame, CA, USA) was used for nuclear staining and images were captured with a Leica Confocal microscope TCS SP5 II (Leica Microsystems, Mannheim, Germany). Hypothalamic areas were defined according to the Paxinos and Watson rat brain atlas [24]. Images were acquired in high (400×) magnification.

### 2.8. Hormone Measurements

Trunk blood was collected two hours before lights off and plasma was extracted with heparin and stored at −80 °C for corticosterone determination with a commercially available ELISA kit (Cat. #402810; Neogen, Lexington, KY, USA). For urine collection, rats were placed in a metabolic cage during the dark phase. Urine was allowed to accumulate overnight in a glass tube placed under the cages. Urine samples were used for 6-sulfatoxi-melatonin (6-S-Mel) determination using a commercially available ELISA kit (Cat. #RE54031; IBL International, Hamburg, Germany).

### 2.9. RNA Extraction and Real Time pCR

Fragments of liver were homogenized in Trizol (50 mg/mL) and the total RNA was extracted and quantified using a Nanodrop 8000 device (Thermo Scientific, Wilmington, DE, USA). The cDNA was synthesized using 2 μg of the total RNA and the High-Capacity cDNA Reverse Transcription Kit (Cat. 4368813). PCR was performed for cytochrome P450 1A2 (*cyp1a2*) and cytosolic sulfotransferase 1A1 (*sult1a1*) genes using the respective Taqman primers assays rn00561082_m1 and rn01510633_m1 and the Taqman Gene Expression Master Mix (Cat. 4369016) in a Step One Plus sequence detection system (Applied Biosystems, Carlsbad, CA, USA). All reagents for reverse transcription and PCR were obtained from Applied Biosystems. Values of mRNA expression were normalized to *gapdh* (rn99999916) gene expression using the ΔΔCT method.

### 2.10. Statistical Analysis

The results are presented as the mean ± standard error of the mean Comparisons were performed using one-way Analysis of Variance followed by Tukey–Kramer post hoc testing or Student’s *t*-test (version, GraphPad Prism Software, Inc., San Diego, CA, USA). Values of *p* < 0.05 indicate a significant difference.

## 3. Results

### 3.1. Metabolic and Endocrine Changes in Rats Exposed to Fructose Consumption during the Light or the Dark Phases

**Figure 1:**
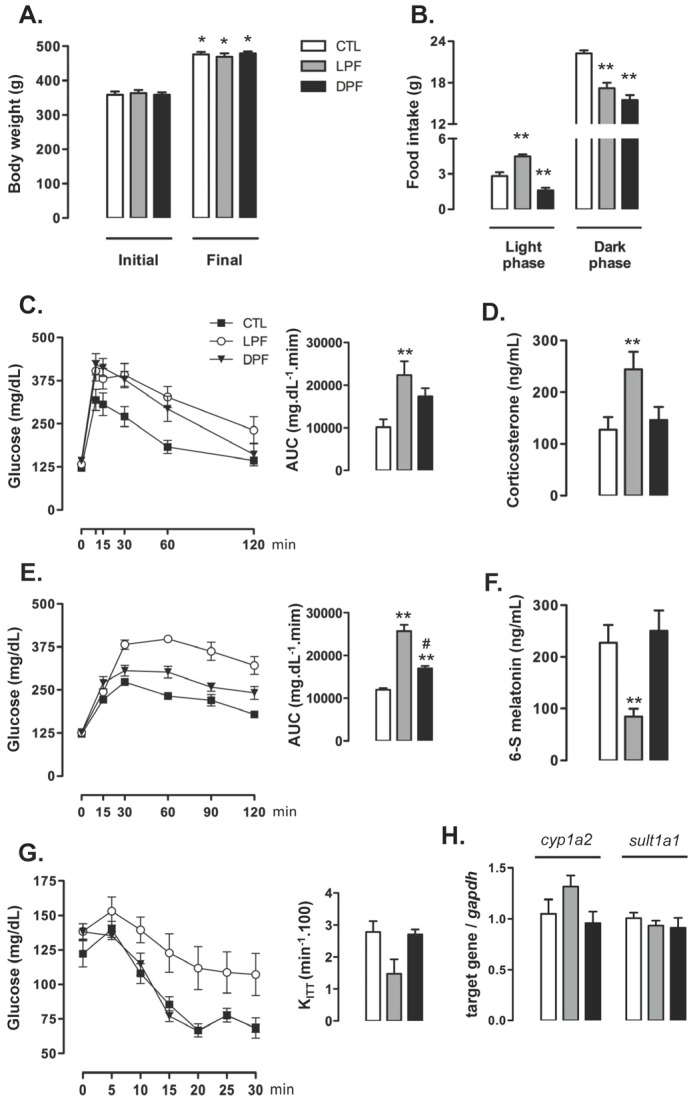
Metabolic and endocrine changes in rats exposed to fructose consumption during the light or the dark phases. Rats assigned to the groups control (CTL), Light Phase Fructose (LPF) and Dark Phase Fructose (DPF) had their (**A**) body weights assessed before and after (8 weeks) treatments. (**B**) Food intake during the light and the dark phases were also assessed at the end of the treatments. After these measurements, the rats were subjected to (**C**) glucose tolerance tests, (**E**) pyruvate tolerance tests and (**G**) insulin tolerance tests. Tests were performed two hours before “lights off” and the area under the curve (AUC) was calculated. Euthanasia was performed two hours before “lights off” when fragments of liver and plasma were collected. (**D**) Plasma samples were used for the determination of corticosterone levels. (**F**) Urine was collected overnight before euthanasia for determination of 6-S-Mel concentration. Fragments of liver were used for relative determination of (**H**) *cyp1a2* and *sult1a1* mRNAs by real time PCR. The results are presented as the means ± standard error of the mean. * *p* < 0.05 vs. the same group before treatment; ** *p* < 0.05 vs. CTL at the same phase of the light/dark cycle; # *p* < 0.05 vs. LPF at the same phase of the light/dark cycle.

Body weights of control CTL, LPF and DPF rats were similar at baseline, prior to the beginning of treatments. Body weight gain after the eight weeks of treatment did not differ among the experimental groups so that the final body weight was similar among rats assigned to the CTL, LPF and DPF groups (Figure 1A). Food intake was also assessed at the eighth week of treatment. The rats exhibited an expected nocturnal eating pattern so that, for every group, food intake during the dark phase was higher than that during the light phase (*p* < 0.001). Consumption of chow during the light phase, however, was increased in LPF rats (58% higher than CTL values; *p* < 0.001), but reduced in DPF rats (44% lower than CTL values; *p* < 0.01). Chow consumption during the dark phase was similarly reduced in LPF and DPF rats (23 and 31% lower than CTL, respectively; *p* < 0.01) (Figure 1B).

Increased glucose levels were found in LFP rats, but not in DFP rats, as evidenced by increased AUC values obtained from the GTTs (120% higher than CTL; *p* < 0.05) (Figure 1C). Conversely, the AUC values obtained from the curve glycemia vs. time after a pyruvate load were increased in both LPF and DPF rats (respectively, 114% and 42% higher than CTL; *p* < 0.05). The values of DPF were, however, 44% lower than those of LFP (*p* < 0.001) (Figure 1E). Apart from these changes in glucose levels during the GTT, our data revealed that neither LPF nor DPF rats exhibited insulin resistance as shown by similar K_ITT_ values (Figure 1G). Endocrine changes in LPF rats were hallmarked by increased levels of corticosterone (91% higher than CTL; *p* < 0.05) and reduced urinary levels of 6-S-Mel (63% lower than CTL; *p* < 0.05) (Figure 1D,F, respectively). Importantly, the expressions of the two main enzymes responsible for hepatic melatonin metabolism, *cyp1a2* and *sult1a1*, were similarly expressed in the liver of CTL, LPF and DPF at the end of treatment (Figure 1H). Thus, decreased urinary levels of 6-S-Mel are likely to result from reduced melatonin production rather than changes in hepatic melatonin metabolism.

Liquid ingestion (either water or 10% fructose) was also assessed. LPF rats ingested less water during the dark phase (19.5 ± 0.8 mL) as compared to CTL (26.2 ± 1.2 mL) (*p* < 0.05; *n* = 5). LPF rats, however, increased liquid ingestion during the light phase. LPF rats consumed a mean volume of 26.02 ± 4.1 mL of 10% fructose while CTL consumed a mean volume of 4.2 ± 0.4 mL of water during the light phase (*p* < 0.05; *n* = 5).

DPF increased liquid ingestion during the dark phase as compared to CTL (39.6 ± 1.2 mL of 10% fructose vs. 26.2 ± 1.2 mL of water; *p* < 0.05; *n* = 5). Water ingestion during the light phase, however, was similar between CTL and DPF.

### 3.2. Hypothalamic AM pK Phosphorylation in Rats Exposed to Fructose Consumption during the Light or the Dark Phases

**Figure 2:**
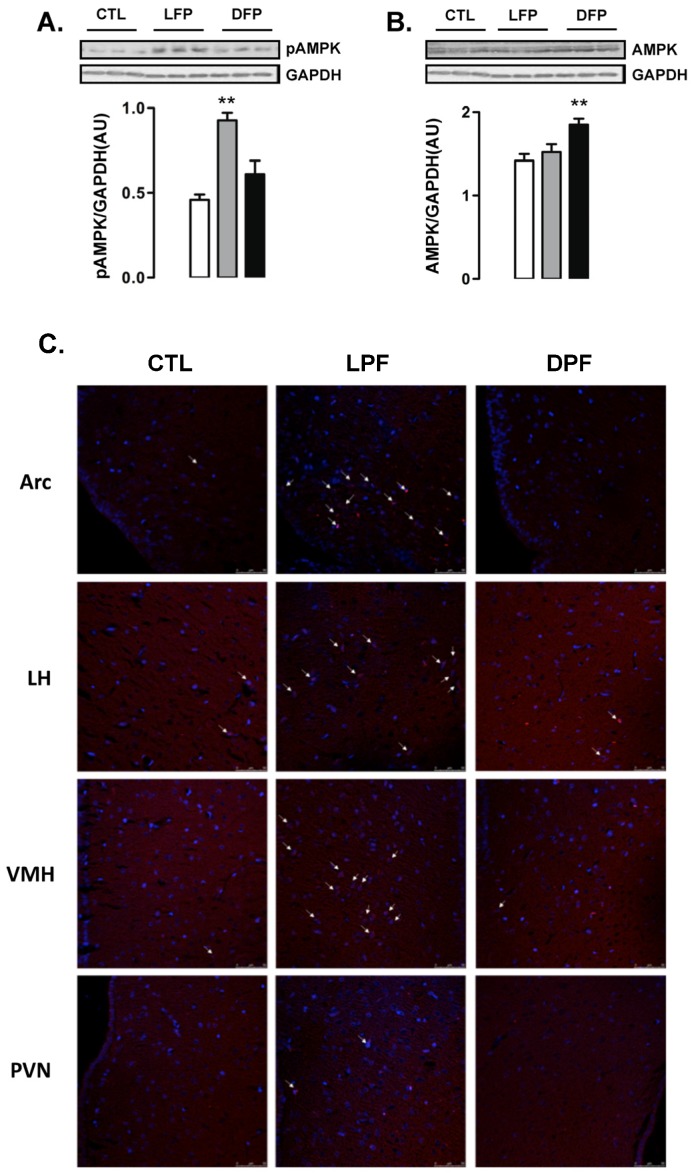
AMPK phosphorylation and content in hypothalamus of rats exposed to fructose consumption during the light or the dark phases. Rats assigned to the groups control (CTL), Light Phase Fructose (LPF) and Dark Phase Fructose (DPF) had their hypothalamus removed at the end of the eighth week of treatment. A first set of samples was used for (**A**) Western blot detection of phosphorylated Adenosine Monophosphate-activated protein kinase (AMPK) and (**B**) total AMPK. Target proteins were normalized to Glyceraldehyde 3-phosphate dehydrogenase (GAPDH). A second set of samples was processed for immunofluorescent staining. Sections were stained using an anti-pAMPK antibody followed by secondary antibody conjugated to Alexafluor 546 (red). Nuclear structures are visualized by 4′,6-diamidino-2-phenylindole (DAPI) probing (Blue). (**C**) Large magnification (400×) images are shown from the arcuate nucleus (ARC), lateral hypothalamus (LH), ventro medial hypothalamus (VMH) and paraventricular nucleus (PVN). The results are presented as the means ± standard error of the mean. ** *p* < 0.05 vs. CTL.

Western blot analyses of whole hypothalamus revealed that the levels of AMPK phosphorylation were increased in LPF but not in DPF rats (102% higher than CTL; *p* < 0.05) (Figure 2A). In contrast, the content of AMPK was not modulated in the hypothalamus of LPF rats but was increased in the hypothalamus of DPF (30% higher than CTL; *p* < 0.05) (Figure 2B).

Immunofluorescent staining was performed to identify the hypothalamic areas that could account for the results seen in the Western blot experiments. We found that the number of cells with phosphorylated AMPK was evidently increased in the regions of the arcuate nucleus (ARC) and the ventro medial hypothalamus (VMH) from LPF rats. No evident of increase in the number of cells with phosphorylated AMPK was found in the regions of the lateral hypothalamus (LH) and the paraventricular nucleus (PVN). In agreement with the Western blot data, we found a similar number of cells with phosphorylated AMPK in the ARC, VMH, LH and PVN regions of the CTL and DPF rats (Figure 2C).

### 3.3. Increased Hypothalamic AMPK Phosphorylation in LPF Phase Advances Food Intake Resulting in Metabolic and Endocrine Changes

Hypothalamic AMPK activation was already reported to stimulate food intake and reduce melatonin production [25,26]. Thus, we decided to assess the relevance of the increased hypothalamic AMPK phosphorylation for LPF rats in a set of experiments in which they received icv injections with Compound C, a pharmacological AMPK inhibitor. The rats were assigned to four different groups in these experiments, as follows: CTL (rats that did not receive fructose and were treated with vehicle icv), LPF (rats that received fructose during the light phase and were treated with vehicle icv), Compound C (CC) (rats that did not receive fructose and were treated with Compound C icv) and LPF/CC (rats that received fructose during the light phase and were treated with Compound C icv). Icv treatments lasted for five days during the eighth week of treatment with fructose.

**Figure 3:**
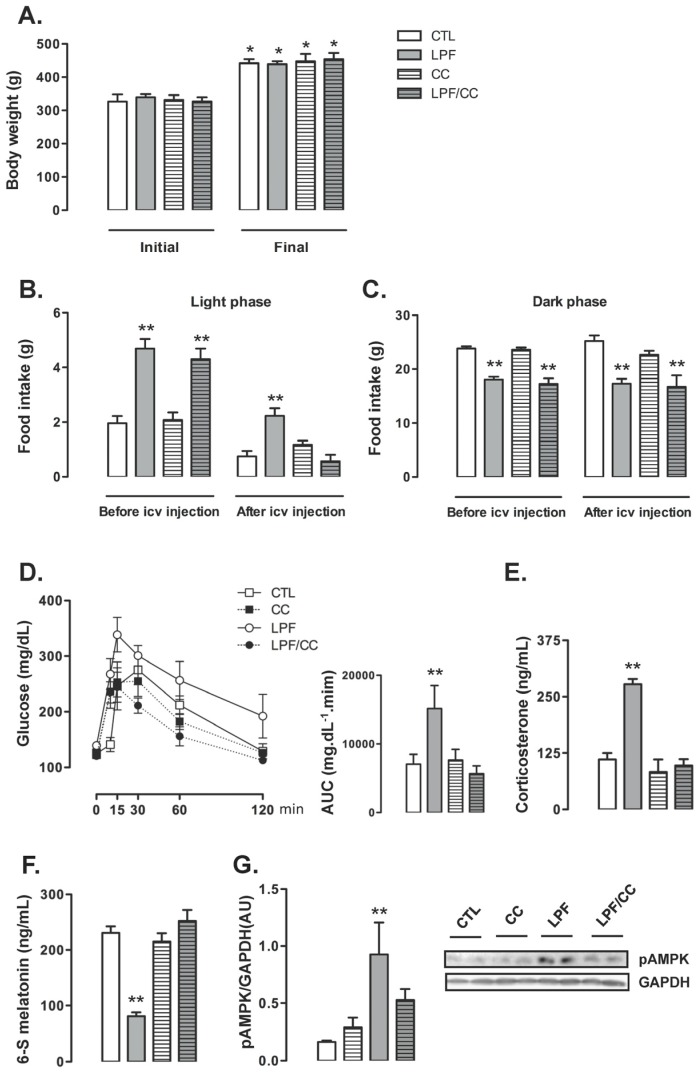
Pharmacological inhibition of adenosine monophosphate-activated protein kinase (AMPK) in the central nervous system of LPF rats. Rats were assigned to the groups control (CTL), Light Phase Fructose (LPF), Compound C (CC) and Light Phase Fructose with Compound C (LPF/CC). Cannula implantation and icv treatments (five days) occurred during the sixth and eighth weeks of fructose treatment, respectively. (**A**) Body weights were assessed before and after (8 weeks) fructose treatment. Food intake was assessed before and after icv injections during the last week of fructose treatment. Data were acquired separately during the (**B**) light and the (**C**) dark phases. After these measurements (**D**), the rats were subjected to glucose tolerance tests. Tests were performed two hours before “lights off” and area under the curve (AUC) was calculated. Euthanasia was performed two hours before lights off, and the hypothalamus and plasma were collected. (**E**) Plasma samples were used for corticosterone determinations. (**F**) Urine was collected overnight before euthanasia for determination of 6-S-Mel concentration. (**G**) Hypothalamus samples were used for Western blot detection of phosphorylated AMPK and normalization by Glyceraldehyde 3-phosphate dehydrogenase (GAPDH). The results are presented as the means ± standard error of the mean. * *p* < 0.05 vs. same group before fructose treatment; ** *p* < 0.05 vs. CTL at the same moment of icv treatment.

Icv treatments did not interfere with the final body weight so that similar values were obtained among the groups at the end of the eighth week of treatment. For the four groups, the final body weight reached values higher than in the beginning of treatments (*p* < 0.05) (Figure 3A). Before the beginning of icv treatments, food intake during the light phase was increased in animals assigned to both LPF and LPF/CC groups (139% and 119% higher than CTL, respectively; *p* < 0.05). Thus, surgical implantation of the cannula in the lateral ventricle alone did not affect the response to light phase fructose described in the previous experiments. After icv treatments, the LPF animals treated with vehicle, but not those treated with Compound C, maintained increased values of food intake during the light phase (200% higher than CTL after treatment with vehicle; *p* < 0.05) (Figure 3B).

Food intake during the dark phase was similarly not affected by cannula implantation per se so that, before icv treatments, the amount of chow ingested by LPF and LPF/CC groups during the dark phase was lower than that ingested by rats assigned to the CTL group (25% and 28% lower than CTL before icv treatment; *p* < 0.05). After icv treatments, food intake during the dark phase remained reduced in both LPF and LPF/CC groups (32% and 34% lower than CTL after icv treatment with vehicle; *p* < 0.05) (Figure 3C).

Glucose tolerance test performed after icv treatments showed that these injections with vehicle did not interfere with the metabolic effect of fructose consumption during the light phase. In these experiments, as in those described above, LPF animals treated with vehicle presented increased glucose levels as evidenced by the AUC values (114% higher than in CTL after icv treatment with vehicle; *p* < 0.05). When compared with CTL rats, the CC and the LPF/CC rats exhibited similar changes in glucose levels during the GTT (evidenced by similar values of AUC) (Figure 3D).

Corticosterone levels were increased in LPF rats treated with vehicle via icv (150% higher than in CTL treated with vehicle; *p* < 0.05). Corticosterone levels of LPF/CC and CC rats remained similar to those of CTL rats after icv treatments (Figure 3E). The urinary 6-S-Mel concentration in LPF rats, but not in LPF/CC and CC rats, was reduced after icv treatments (65% lower than CTL after icv treatment with vehicle; *p* < 0.05) (Figure 3F). The phosphorylation levels of hypothalamic AMPK were increased in LPF rats after treatment with vehicle (460% higher than in CTL treated with vehicle; *p* < 0.05). Treatment with Compound C icv blunted this response so that hypothalamic AMPK phosphorylation in LPF/CC rats was similar to that of CTL rats (Figure 3G).

Pharmacological inhibition of hypothalamic AMPK in LPF rats was able to blunt both the reduction in urinary 6-S-Mel levels and the changes hallmarked by increased corticosterone and increased glucose levels during the GTT. However, pharmacological AMPK inhibition in LPF also inhibited the shift in spontaneous chow ingestion to the light phase.

### 3.4. Increased Food Intake during the Light Phase Seen in LPF Contributes to Metabolic and Endocrine Changes

To investigate if the increase in food intake during the light phase was involved in the metabolic and endocrine changes observed in LPF animals, we designed an experimental protocol in which the animals were assigned to four different groups: CTL (rats that consumed chow ad libitum and did not receive fructose), LPF (rats that consumed chow ad libitum and received fructose during the light phase), Chow-R (rats that consumed chow exclusively during the dark phase and did not receive fructose) and LPF/Chow-R (rats that consumed chow exclusively during the dark phase and received fructose during the light phase).

**Figure 4:**
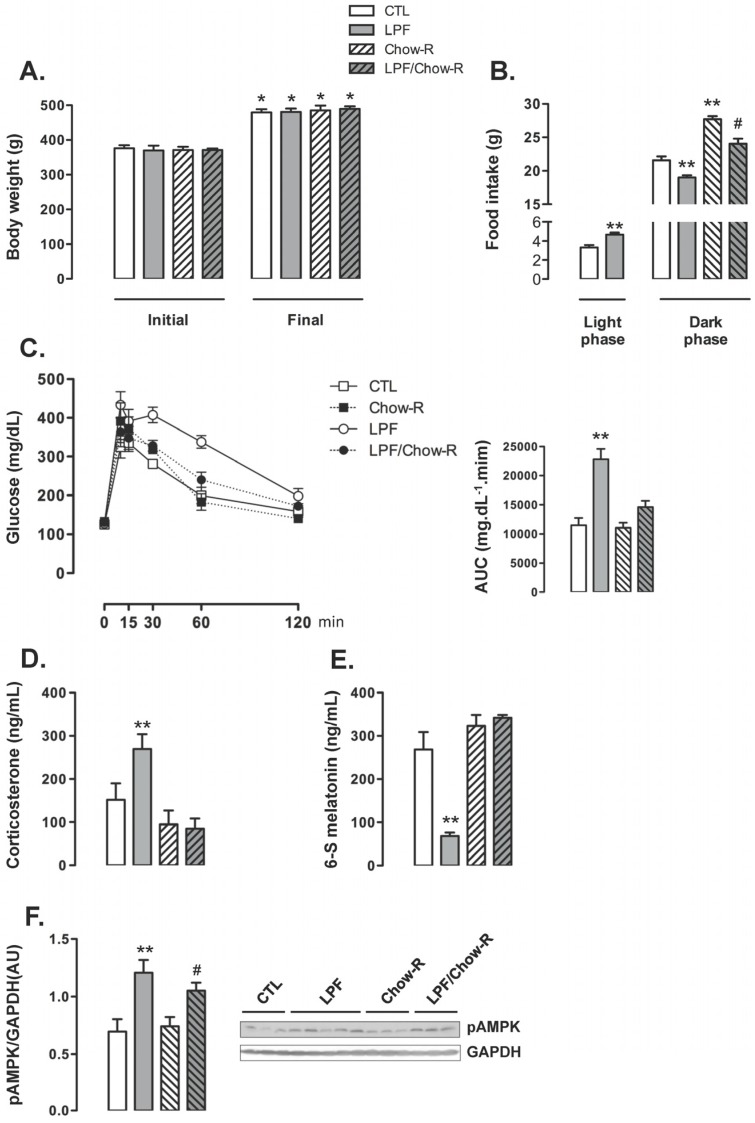
Dark-restricted feeding in rats exposed to fructose during the light phase. Rats were assigned to the groups Control (CTL), Light Phase Fructose (LPF), Chow restriction to the dark phase (Chow-R) and Light Phase Fructose with Chow restriction to the dark phase (LPF/Chow-R). (**A**) Body weights were assessed before and after (eight week) treatments; (**B**) Food intake during the light and the dark phases were also assessed at the end of the treatments; After these measurements (**C**), the rats were subjected to glucose tolerance tests. Tests were performed two hours before “lights off” and area under the curve (AUC) was calculated. Euthanasia was performed two hours before “lights off” and the hypothalamus and plasma were collected; (**D**) Plasma samples were used for the determination of corticosterone levels; (**E**) Urine was collected overnight before euthanasia for determination of the 6-S-Mel concentration; (**F**) Hypothalamus samples were used for Western blot detection of phosphorylated adenosine monophosphate-activated protein kinase and normalization by Glyceraldehyde 3-phosphate dehydrogenase (GAPDH). The results are presented as the means ± standard error of the mean. * *p* < 0.05 vs. same group before treatment; ** *p* < 0.05 vs. CTL at the same phase of the light/dark cycle; # *p* < 0.05 vs. Chow-R at the same phase of the light/dark cycle.

The body weight of animals assigned to the four groups were similar at the beginning of the treatments. The four groups of animals exhibited a similar increase in body weight so that absolute body weights at the end of the treatments were also similar among the groups (Figure 4A). The increase in food intake during the light phase exhibited by LPF rats was replicated in this set of experiments (40% higher than food intake of CTL during the light phase; *p* < 0.05). The food intake during the dark phase was increased in Chow-R compared with CTL rats (26% higher; *p* < 0.05). Apart from that, consumption of fructose during the light phase resulted in reduced food intake during the dark phase irrespective of food restriction to this phase. Thus, food intake during the dark phase was reduced in LPF/Chow-R (14% lower than in Chow-R; *p* < 0.05) and in LPF (12% lower than in CTL; *p* < 0.05) (Figure 4B).

LPF animals exhibited consistent increased glucose levels during the GTT as evidenced by the AUC values (98% higher than CTL; *p* < 0.05). The glucose levels during the GTT were not modulated in Chow-R compared with CTL animals. Interestingly, increased glucose levels during the GTT were not replicated in LPF/Chow-R rats (Figure 4C). With regard to the endocrine profile, Chow-R exhibited corticosterone and urinary 6-S-Mel concentrations similar to those of CTL rats. The increase in circulating corticosterone levels (77%; *p* < 0.05) and the reduction in the urinary concentration of 6-S-Mel (74%; *p* < 0.05) observed in LPF rats when compared with CTL rats were not detected in LPF/Chow-R (respectively, Figure 4D,E). The hypothalamic AMPK phosphorylation levels observed in CTL and in LPF rats were not affected by restricting food availability to the dark phase. Thus, the amounts of phosphorylated AMPK were increased in LPF compared with CTL rats (72%; *p* < 0.05) and in LPF/Chow-R compared with Chow-R rats (41%; *p* < 0.05) (Figure 4F).

### 3.5. Reduced Melatonin Production in LPF Rats Leads to Increased Corticosterone Levels and Glucose Intolerance

To determine the metabolic relevance for reduced 6-S-Mel levels in LPF rats, we designed the next experimental protocol in which the animals were assigned to the following four different groups: CTL (rats that did not receive either fructose or melatonin), LPF (rats that received fructose during the light phase), Mel (rats that received melatonin exclusively during the dark phase) and LPF/Mel (rats that received fructose during the light phase and melatonin exclusively during the dark phase).

**Figure 5:**
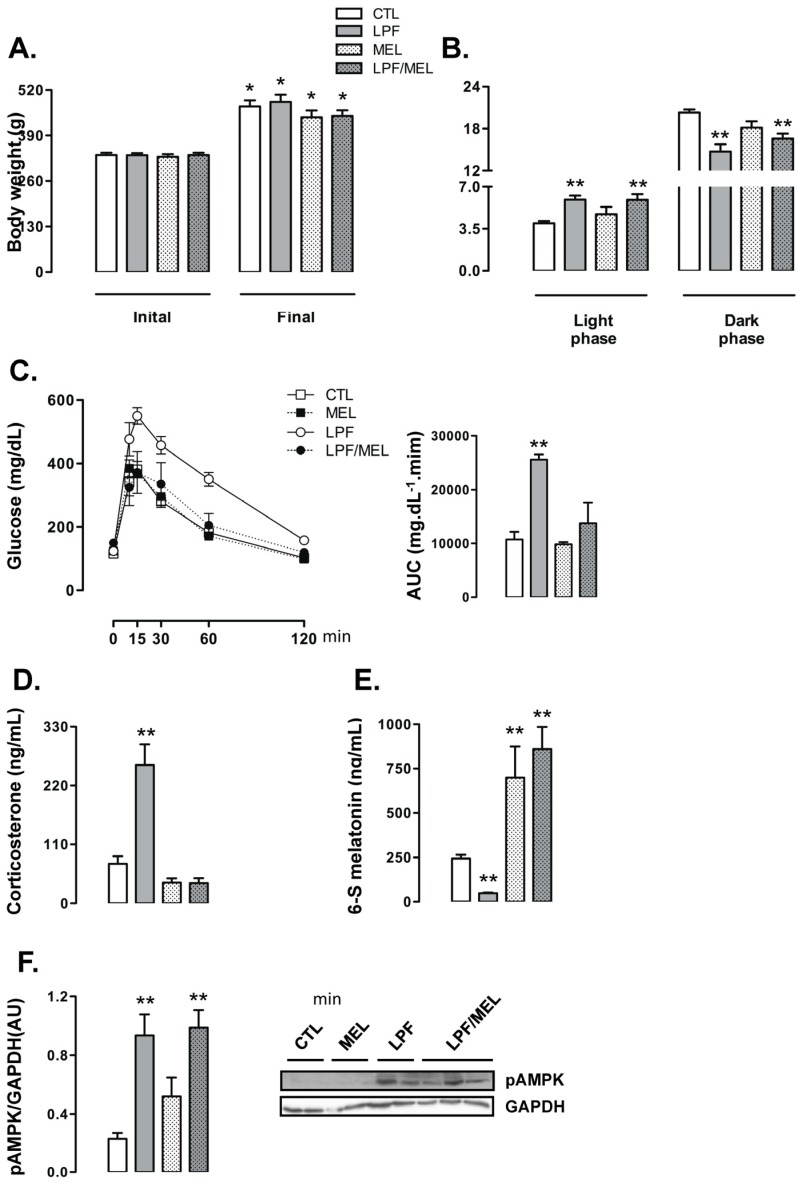
Nocturnal melatonin administration in rats exposed to fructose during the light phase. Rats were assigned to the groups control (CTL), Light Phase Fructose (LPF), melatonin (Mel) and Light Phase Fructose with melatonin (LPF/Mel). (**A**) Body weights were assessed before and after (eight week) treatments; (**B**) Food intake during the light and the dark phases were also assessed at the end of the treatments; After these measurements (**C**), the rats were subjected to glucose tolerance tests. Tests were performed two hours before “lights off” and area under the curve (AUC) was calculated. Euthanasia was performed two hours before “lights off” and the hypothalamus and plasma were collected; (**D**) Plasma samples were used for the determination of corticosterone levels; (**E**) Urine was collected overnight before euthanasia for determination of 6-S-Mel concentration; (**F**) Hypothalamus samples were used for Western blot detection of phosphorylated adenosine monophosphate-activated protein kinase and normalization by Glyceraldehyde 3-phosphate dehydrogenase (GAPDH). The results are presented as the means ± standard error of the mean. * *p* < 0.05 vs. same group before treatment; ** *p* < 0.05 vs. CTL at the same phase of the light/dark cycle.

When compared to their initial body weights, the rats belonging to the four experimental groups exhibited increased body weight at the end of the treatment (*p* < 0.05). Treatment with melatonin did not affect the changes in body weights throughout the experimental period so that final body weights of the rats belonging to the CTL, LPF, Mel and LPF/Mel groups were similar (Figure 5A). The changes in the food intake profile observed in LPF animals, hallmarked by increased values during the light phase and reduced values during the dark phase, were not altered by melatonin treatment. Additionally, melatonin treatment per se did not affect food intake. Thus, food intake by both LPF and LPF/Mel rats was increased during the light phase (approximately 50% higher than CTL; *p* < 0.05) and reduced during the dark phase (approximately 17% lower than CTL; *p* < 0.05) (Figure 5B).

Treatment with melatonin was able to blunt the increase in glucose levels induced by fructose consumption during the light phase. The AUC values obtained from the GTT were increased in LPF (138% higher than CTL; *p* < 0.05) but not in LPF/Mel rats. In turn, treatment with melatonin in fructose-naive rats did not alter glucose levels during the GTT (AUC values similar to CTL) (Figure 5C). As an example of what was observed for glucose levels along with the GTT, corticosterone levels were increased in LPF (249% higher than CTL; *p* < 0.05) but not in LPF/Mel animals. Melatonin treatment alone did not alter corticosterone concentrations (Figure 5D).

In this set of experiments, we also found that LPF rats had reduced urinary 6-S-Mel concentrations (80% lower than CTL; *p* < 0.05). Fructose consumption during the light phase, however, failed to reduce urinary 6-S-Mel concentrations in rats consuming melatonin (urinary 6-S-Mel concentration are similar between Mel and LPF/Mel groups). The urinary 6-S-Mel concentration was found to be similarly increased in both Mel and LPF/Mel groups (187% and 254% higher than CTL, respectively; *p* < 0.05) (Figure 5E).

Melatonin treatment alone did not interfere with hypothalamic AMPK phosphorylation so that these levels in rats belonging to the Mel group were similar to those of the CTL group. Melatonin treatment was also unable to modulate the increase in hypothalamic AMPK phosphorylation induced by the consumption of fructose during the light phase (LPF and LPF/Mel were, respectively, 302% and 325% higher than CTL; *p* < 0.05) (Figure 5F).

## 4. Discussion

The data presented herein show that rats receiving fructose during the light phase developed increased hypothalamic AMPK phosphorylation, reduced urinary 6-S-Mel, increased chow ingestion during the light phase and impaired glucose tolerance. Importantly, these combined changes were not observed in rats receiving fructose exclusively during the dark phase. Our data supports the conclusion that this shift in food intake is of pivotal relevance for metabolic outcomes because fructose ingestion during the light phase with simultaneous chow restriction to the dark phase fails to impair glucose tolerance. This finding is in accordance with recent publications showing that rats that increase their food intake during the light phase (either by forced activity protocols during the light phase or by simple restriction of food availability during dark phase) become glucose intolerant [27]. Out-of-phase feeding seems also to be relevant for human metabolism as subgroups of diabetic patients who display night eating behavior also have impaired glycemic control based on increased glycated hemoglobin levels [28].

The present data also allow us to conclude that hypothalamic AMPK activation (which takes place mainly in the ARC and VMH) is a key event induced by fructose ingestion during the light phase that increases out-of-phase feeding. This sequential cause/effect relationship is supported by our data which show: 1-pharmacological AMPK inhibition in the central nervous system using Compound C abrogates the shift in food intake and impaired glucose tolerance induced by fructose consumption during the light phase; and 2-fructose consumption during the light phase is still able to induce hypothalamic AMPK phosphorylation in rats for which food availability has been restricted to the dark phase. Accordingly, previous studies have already shown that a consistent increase in food intake occurs when hypothalamic AMPK is activated in the ARC and VMH [25,29] and that fructose metabolism in the hypothalamus results in a rapid reduction in ATP with parallel increase in the AMP/ATP ratio that activates AMPK and stimulates food intake [18,30]. To date, in vitro experiments have also shown that that fructose can directly activate AMPK in hypothalamic GT1-7 cells [31].

Previous publications have also shown that hypothalamic AMPK activation with pharmacological approaches triggers a counter-regulatory response hallmarked by increased EGP. The mechanisms for this response are not completely understood. However, it was demonstrated that hypothalamic AMPK activation in nuclei such as VMH and ARC is able to spread peripheral signals that lead to the secretion of glucocorticoids, glucagon and catecholamines [32,33,34]. These hormones are classically known to act in the liver by increasing gluconeogenesis and glycogenolysis, therefore stimulating EGP. In this context, we have previously demonstrated that intra-cerebro ventricular injections with fructose during the light phase lead to an acute activation of hypothalamic AMPK in the central nervous system and consequently increases corticosterone levels that raise whole-body gluconeogenesis [17].

Increased corticosterone levels as an acute response to an oral fructose load have been formerly demonstrated by other groups [35,36]. The present data add further information to this field by revealing that chronic fructose consumption exclusively during the resting light phase can also increase corticosterone. As an example of glucose levels during the GTT, the increase of corticosterone levels was equally prevented by restricting chow availability to the dark phase and by pharmacological AMPK inhibition in the central nervous system. Thus, our data show that the ability of chronic fructose consumption during the light phase to increase corticosterone levels after eight weeks of treatment relies on the chronically light phase-shifted chow ingestion induced by hypothalamic AMPK activation.

Having established that hypothalamic AMPK activation and increased food intake during the light phase are important for the enhance in corticosterone levels observed in LPF rats, we next explored how changes in the central nervous system result in peripheral endocrine modulations. It was previously demonstrated that exposing Sprague–Dawley rats to a 60% fructose-enriched diet ad libitum resulted in a reduction of the levels of urinary 6-S-Mel [6]. 6-S-Mel is the most abundant melatonin metabolite. Its formation requires the conversion of melatonin into the intermediary 6-hydroxymelatonin that suffers subsequent sulfation. These two reactions (hydroxylation and sulfation) occur predominantly in the liver and, in rats, they are respectively catalyzed by cytochrome P450 1A2 (*cyp1a2*) and cytosolic sulfotransferase 1A1 (*sult1a1*) [37,38,39]. In this context, the determination of 6-S-Mel excretion in the urine is a well-recognized method to estimate melatonin production [40,41].

As LPF developed increased liquid and food intake during the light phase, we can presume that these rats may present a partial shift of global activity to the light phase. It is unlike, however, that increased activity during the light phase per se may account for the reduced melatonin metabolite concentration seen in LPF. This proposition is corroborated by studies showing that forced physical activity (swimming) during the light phase fails to modulate nocturnal melatonin production in rats [42]. We and others have also previously shown that light phase-restricted feeding with standard chow fail to reduce melatonin metabolite in the urine or melatonin circulating levels during the dark phase [43,44]. On the other hand, the daytime consumption of carbohydrates by rodents seems to be particularly relevant to yield reductions in nocturnal melatonin production. It was previously demonstrated that reduced amplitude of nocturnal melatonin levels was only achieved by offering a combination of a carbohydrate-enriched diet and standard chow during the light-resting phase [44]. Accordingly, the present data reveals that the consumption of fructose during the light phase fails to reduce 6-S-Mel production in rats subjected to restriction of chow availability to the dark phase. Our data from the experiments with Compound C further corroborates this hypothesis because the pharmacological inhibition of AMPK in the central nervous system abrogated both the shift in food intake to the light phase observed in LPF rats and the reduction in urinary 6-S-Mel. Importantly, LPF rats did not show any modulation of the hepatic expression of *cyp1a2* and *sult1a1*. Thus, it is unlikely that reduced urinary 6-S-Mel seen in LPF rats resulted from lower hepatic metabolism of melatonin.

The reduction in the concentration of melatonin metabolite seen in the urine of LPF rats cannot be attributed to an increase in water intake during the dark phase that could potentially increase urine volume and dilute melatonin metabolite. This can be concluded because LPF rats actually displayed reduced water intake during the dark phase, the period during which the urine samples were collected.

To date, the negative modulation of melatonin secretion secondary to hypothalamic AMPK activation has already been shown in other species. Menassol et al. demonstrated that acute icv injection with AICAR (a pharmacological AMPK activator) in ewes can actually reduce the amplitude of the nocturnal melatonin surge. This modulation occurred irrespective of changes in the rhythm of melatonin production [26]. As our data suggest, the ability of hypothalamic AMPK activation induced by fructose ingestion during the light phase to reduce melatonin production relies on changes in the rhythm of feeding behavior of the rat. Whether this applies to different species remains to be determined.

The causal relationship between the reduced urinary 6-S-Mel, impaired glucose tolerance and increased corticosterone were further examined in our experiments in which LPF rats were treated with melatonin. We have collected evidence to support the proposition that reduced urinary 6-S-Mel in rats consuming fructose during the light phase is likely to result from reduced melatonin production that is pivotal for the increase in corticosterone levels and impaired glucose tolerance as these adaptations were not observed in LPF rats receiving melatonin. Accordingly, melatonin has already been demonstrated to blunt insulin resistance induced by ad libitum consumption of a 60% fructose enriched diet in Wistar rats [12]. On the other hand, our data revealed that supplementation with melatonin had no effect on food intake. This is in accordance with other studies showing that, although melatonin is able to modulate the expression of orexigenic and anorexigenic neurotransmitters, this hormone has very discreet direct effect on food intake [13,45]. Altogether, our results indicate that increased food intake during the light phase seen in LPF animals is likely to be a cause, rather than a consequence, of reduced urinary 6-S-Mel.

The increased corticosterone levels observed in experimental conditions characterized by reduced melatonin production can be explained by the suppressive action that the pineal hormone exert on the Hypothalamus-Pituitary-Adrenal (HPA) axis. It has already been shown that melatonin acts through MT1 receptors to suppress adrenocorticotropic hormone-induced cortisol production in cultured adrenal glands isolated from primates [46]. This action of melatonin was reported to be dependent on the reduction of intracellular cAMP levels [46]. A similar response was found in adrenal glands from rats cultured with melatonin [47]. It is important to note that the suppressive action of melatonin over the HPA axis might not be restricted to direct action on the adrenal glands because rats treated with melatonin were also shown to have reduced corticotropin-releasing hormone and adrenocorticotropic hormone levels after a stress stimulus [48].

## 5. Conclusions

In summary, the present study demonstrates that fructose consumption during the light phase, but not during the dark phase, results in glucose intolerance and increased corticosterone levels. The effects of daytime consumption of fructose are secondary to hypothalamic AMPK activation that leads to upregulation of food intake during the light phase. We also show that the reduction of urinary 6-sulfatoxymelatonin (6-S-Mel) (probably indicative of reduced melatonin production) due to hypothalamic AMPK activation is a key event that mediates the increase in corticosterone and impaired glucose tolerance induced by the consumption of fructose during the light phase.

## References

[1] American Diabetes Association (2010). Diagnosis and classification of diabetes mellitus. Diabetes Care.

[2] Reaven G.M. (1988). Banting lecture 1988. Role of insulin resistance in human disease. Diabetes.

[3] Oron-herman M., Kamari Y., Grossman E., Yeger G., Peleg E., Shabtay Z., Shamiss A., Sharabi Y. (2008). Metabolic syndrome: Comparison of the two commonly used animal models. Am. J. Hypertens..

[4] Chan S.M., Sun R.Q., Zeng X.Y., Choong Z.H., Wang H., Watt M.J., Ye J.M. (2013). Activation of PPARα ameliorates hepatic insulin resistance and steatosis in high fructose-fed mice despite increased endoplasmic reticulum stress. Diabetes.

[5] Stanhope K.L., Schwarz J.M., Keim N.L., Griffen S.C., Bremer A.A., Graham J.L., Hatcher B., Cox C.L., Dyachenko A., Zhang W. (2009). Consuming fructose-sweetened, not glucose-sweetened, beverages increases visceral adiposity and lipids and decreases insulin sensitivity in overweight/obese humans. J. Clin. Investig..

[6] Leibowitz A., Peleg E., Sharabi Y., Shabtai Z., Shamiss A., Grossman E. (2008). The role of melatonin in the pathogenesis of hypertension in rats with metabolic syndrome. Am. J. Hypertens..

[7] La Fleur S.E., Kalsbeek A., Wortel J., Van Der Vliet J., Buijs R.M. (2001). Role for the pineal and melatonin in glucose homeostasis: Pinealectomy increases night-time glucose concentrations. J. Neuroendocrinol..

[8] Lima F.B., Machado U.F., Bartol I., Seraphim P.M., Sumida D.H., Moraes S.M., Hell N.S., Okamoto M.M., Saad M.J., Carvalho C.R. (1998). Pinealectomy causes glucose intolerance and decreases adipose cell responsiveness to insulin in rats. Am. J. Physiol..

[9] Nogueira T.C., Lellis-Santos C., Jesus D.S., Taneda M., Rodrigues S.C., Amaral F.G., Lopes A.M., Cipolla-Neto J., Bordin S., Anhê G.F. (2011). Absence of melatonin induces night-time hepatic insulin resistance and increased gluconeogenesis due to stimulation of nocturnal unfolded protein response. Endocrinology.

[10] Wolden-Hanson T., Mitton D.R., McCants R.L., Yellon S.M., Wilkinson C.W., Matsumoto A.M., Rasmussen D.D. (2000). Daily melatonin administration to middle-aged male rats suppresses body weight, intraabdominal adiposity, and plasma leptin and insulin independent of food intake and total body fat. Endocrinology.

[11] Shieh J.M., Wu H.T., Cheng K.C., Cheng J.T. (2009). Melatonin ameliorates high fat diet-induced diabetes and stimulates glycogen synthesis via a PKCzeta-Akt-GSK3beta pathway in hepatic cells. J. Pineal Res..

[12] Kitagawa A., Ohta Y., Ohash K. (2012). Melatonin improves metabolic syndrome induced by high fructose intake in rats. J. Pineal Res..

[13] Cano B.P., Pagano E.S., Jiménez-Ortega V., Fernández-Mateos P., Esquifino A.I., Cardinali D.P. (2014). Melatonin normalizes clinical and biochemical parameters of mild inflammation in diet-induced metabolic syndrome in rats. J. Pineal Res..

[14] Arble D.M., Bass J., Laposky A.D., Vitaterna M.H., Turek F.W. (2009). Circadian timing of food intake contributes to weight gain. Obesity.

[15] Hatori M., Vollmers C., Zarrinpar A., DiTacchio L., Bushong E.A., Gill S., Leblanc M., Chaix A., Joens M., Fitzpatrick J.A. (2012). Time-restricted feeding without reducing caloric intake prevents metabolic diseases in mice fed a high-fat diet. Cell Metab..

[16] Morris M., Araujo I.C., Pohlman R.L., Marques M.C., Rodwan N.S., Farah V.M. (2012). Timing of fructose intake: An important regulator of adiposity. Clin. Exp. Pharmacol. Physiol..

[17] Kinote A., Faria J.A., Roman E.A., Solon C., Razolli D.S., Ignacio-Souza L.M., Sollon C.S., Nascimento L.F., de Araújo T.M., Barbosa A.P. (2012). Fructose-induced hypothalamic AMPK activation stimulates hepatic PEPCK and gluconeogenesis due to increased corticosterone levels. Endocrinology.

[18] Cha S.H., Wolfgang M., Tokutake Y., Chohnan S., Lane M.D. (2008). Differential effects of central fructose and glucose on hypothalamic malonyl-CoA and food intake. Proc. Natl. Acad. Sci. USA.

[19] Stunkard A.J., Grace W.J., Wolff H.G. (1955). The night-eating syndrome: A pattern of food intake among certain obese patients. Am. J. Med..

[20] Rand C.S., Macgregor A.M., Stunkard A.J. (1997). The night eating syndrome in the general population and among postoperative obesity surgery patients. Int. J. Eat. Disord..

[21] Gallant A., Drapeau V., Allison K.C., Tremblay A., Lambert M., O'Loughlin J., Lundgren J.D. (2014). Night eating behavior and metabolic heath in mothers and fathers enrolled in the QUALITY cohort study. Eat. Behav..

[22] Birketvedt G.S., Florholmen J., Sundsfjord J., Osterud B., Dinges D., Bilker W., Stunkard A. (1999). Behavioral and neuroendocrine characteristics of the night-eating syndrome. JAMA.

[23] Lin Q.M., Zhao S., Zhou L.L., Fang X.S., Fu Y., Huang Z.T. (2013). Mesenchymal stem cells transplantation suppresses inflammatory responses in global cerebral ischemia: Contribution of TNF-α-induced protein 6. Acta Pharmacol. Sin..

[24] Paxinos G., Watson C. (1997). The Rat Brain in Stereotaxic Coordinates.

[25] Kim E.K., Miller I., Aja S., Landree L.E., Pinn M., McFadden J., Kuhajda F.P., Moran T.H., Ronnett G.V. (2004). C75, a fatty acid synthase inhibitor, reduces food intake via hypothalamic AMP-activated protein kinase. J. Biol. Chem..

[26] Menassol J.B., Tautou C., Collet A., Chesneau D., Lomet D., Dupont J., Malpaux B., Scaramuzzi R.J. (2011). The effect of an intracerebroventricular injection of metformin or AICAR on the plasma concentrations of melatonin in the ewe: Potential involvement of AMPK?. BMC Neurosci..

[27] Salgado-Delgado R.C., Saderi N., Basualdo M del C., Guerrero-Vargas N.N., Escobar C., Buijs R.M. (2013). Shift work or food intake during the rest phase promotes metabolic disruption and desynchrony of liver genes in male rats. PLoS ONE.

[28] Hood M.M., Reutrakul S., Crowley S.J. (2014). Night eating in patients with type 2 diabetes. Associations with glycemic control, eating patterns, sleep, and mood. Appetite.

[29] Namkoong C., Kim M.S., Jang P.G., Han S.M., Park H.S., Koh E.H., Lee W.J., Kim J.Y., Park I.S., Park J.Y. (2005). Enhanced hypothalamic AMP-activated protein kinase activity contributes to hyperphagia in diabetic rats. Diabetes.

[30] Lane M.D., Cha S.H. (2009). Effect of glucose and fructose on food intake via malonyl-CoA signaling in the brain. Biochem. Biophys. Res. Commun..

[31] Burmeister M.A., Ayala J., Drucker D.J., Ayala J.E. (2013). Central glucagon-like peptide 1 receptor-induced anorexia requires glucose metabolism-mediated suppression of AMPK and is impaired by central fructose. Am. J. Physiol. Endocrinol. Metab..

[32] Mccrimmon R.J., Shaw M., Fan X., Cheng H., Ding Y., Vella M.C., Zhou L., McNay E.C., Sherwin R.S. (2008). Key role for AMP-activated protein kinase in the ventromedial hypothalamus in regulating counterregulatory hormone responses to acute hypoglycemia. Diabetes.

[33] Han S.M., Namkoong C., Jang P.G., Park I.S., Hong S.W., Katakami H., Chun S., Kim S.W., Park J.Y., Lee K.U. (2005). Hypothalamic AMP-activated protein kinase mediates counter-regulatory responses to hypoglycaemia in rats. Diabetologia.

[34] Alquier T., Kawashima J., Tsuji Y., Kahn B.B. (2007). Role of hypothalamic adenosine 5'-monophosphate-activated protein kinase in the impaired counterregulatory response induced by repetitive neuroglucopenia. Endocrinology.

[35] Brindley D.N., Cooling J., Glenny H.P., Burditt S.L., McKechnie I.S. (1981). Effects of chronic modification of dietary fat and carbohydrate on the insulin, corticosterone and metabolic responses of rats fed acutely with glucose, fructose or ethanol. Biochem. J..

[36] Brindley D.N., Saxto J., Shahidullah H., Armstrong M. (1985). Possible relationships between changes in body weight set-point and stress metabolism after treating rats chronically with D-fenfluramine. Effects of feeding rats acutely with fructose on the metabolism of corticosterone, glucose, fatty acids, glycerol and triacylglycerol. Biochem. Pharmacol..

[37] Skene D.J., Papagiannidou E., Hashemi E., Snelling J., Lewis D.F., Fernandez M., Ioannides C. (2001). Contribution of CYP1A2 in the hepatic metabolism of melatonin: Studies with isolated microsomal preparations and liver slices. J. Pineal Res..

[38] Semak I., Korik E., Antonova M., Wortsman J., Slominski A. (2008). Metabolism of melatonin by cytochrome P450s in rat liver mitochondria and microsomes. J. Pineal Res..

[39] Honma W., Kamiyama Y., Yoshinari K., Sasano H., Shimada M., Nagata K., Yamazoe Y. (2001). Enzymatic characterization and interspecies difference of phenol sulfotransferases, ST1A forms. Drug Metab. Dispos..

[40] Pääkkönen T., Mäkinen T.M., Leppäluoto J., Vakkuri O., Rintamäki H., Palinkas L.A., Hassi J. (2006). Urinary melatonin: A noninvasive method to follow human pineal function as studied in three experimental conditions. J. Pineal Res..

[41] Stieglitz A., Spiegelhalter F., Klante G., Heldmaier G. (1995). Urinary 6-sulphatoxymelatonin excretion reflects pineal melatonin secretion in the Djungarian hamster (Phodopus sungorus). J. Pineal Res..

[42] Golombek D.A., Burin L., Cardinali D.P. (1992). Time-dependency for the effect of different stressors on rat pineal melatonin content. Acta Physiol. Pharmacol. Ther. Latinoam..

[43] De Almeida Faria J., de Araújo T.M., Mancuso R.I., Meulman J., da Silva Ferreira D., Batista T.M., Vettorazzi J.F., da Silva P.M., Rodrigues S.C., Kinote A. (2016). Day-restricted feeding during pregnancy and lactation programs glucose intolerance and impaired insulin secretion in male rat offspring. Acta Physiol..

[44] Selmaoui B., Oguine A., Thibault L. (2001). Food access schedule and diet composition alter rhythmicity of serum melatonin and pineal NAT activity. Physiol. Behav..

[45] Ríos-Lugo M.J., Jiménez-Ortega V., Cano-Barquilla P., Mateos P.F., Spinedi E.J., Cardinali D.P., Esquifino A.I. (2015). Melatonin counteracts changes in hypothalamic gene expression of signals regulating feeding behavior in high-fat fed rats. Horm. Mol. Biol. Clin. Investig..

[46] Torres-Farfan C., Richter H.G., Rojas-García P., Vergara M., Forcelledo M.L., Valladares L.E., Torrealba F., Valenzuela G.J., Serón-Ferré M. (2003). mt1 Melatonin receptor in the primate adrenal gland: Inhibition of adrenocorticotropin-stimulated cortisol production by melatonin. J. Clin. Endocrinol. Metab..

[47] Richter H.G., Torres-Farfan C., Garcia-Sesnich J., Abarzua-Catalan L., Henriquez M.G., Alvarez-Felmer M., Gaete F., Rehren G.E., Seron-Ferre M. (2008). Rhythmic expression of functional MT1 melatonin receptors in the rat adrenal gland. Endocrinology.

[48] Konakchieva R., Mitev Y., Almeida O.F., Patchev V.K. (1997). Chronic melatonin treatment and the hypothalamo-pituitary-adrenal axis in the rat: Attenuation of the secretory response to stress and effects on hypothalamic neuropeptide content and release. Biol. Cell.

